# Epicardial adipose tissue thickness assessed by cardiac magnetic resonance is an independent indicator for coronary artery stenosis in asymptomatic type 2 diabetic patients

**DOI:** 10.1186/1532-429X-14-S1-P78

**Published:** 2012-02-01

**Authors:** Hee Yeong Kim, Young Jin Kim, Byoung Wook Choi

**Affiliations:** 1 Department of Radiology, Severance Hospital, University College of Medicine, Seoul, Republic of Korea

## Background

We investigated the association between epicardial adipose tissue (EAT) thickness and myocardial ischemia as well as coronary artery stenosis assessed by cardiovascular magnetic resonance (CMR) in asymptomatic type 2 diabetic patients.

## Methods

A total of 100 type 2 diabetic subjects (51 men and 49 women; mean age: 56.4 ± 7.6 years) were enrolled. Silent myocardial ischemia by CMR was defined as an evidence of inducible ischemia or myocardial infarction and signal reduction or stenosis of ≥ 50% in the vessel diameter were used as the criteria for significant coronary artery stenosis on coronary MR angiography.

## Results

EAT thickness was positively correlated with BMI, waist-to-hip ratio, systolic blood pressure, postprandial glucose, fasting/postprandial triglyceride, HbA1c level, and HOMA-IR. A total of 24 patients had significant coronary artery stenosis and 14 patients had silent myocardia ischemia in CMR (3 with silent myocardial infarction, 13 with inducible ischemia, 2 with both). EAT thickness was higher in patients who had significant stenosis; however, it was not different between the subjects with silent myocardial ischemia and the subjects with no evidence of silent myocardial ischemia in CMR (13.0 ± 2.6 mm vs. 11.5 ± 2.1 mm, p=0.01, 12.8 ± 2.1 vs. 11.7 ± 2.3 mm, p=0.11, respectively). In multivariate logistic regression analysis, EAT thickness was an independent indicator for significant coronary artery stenosis after adjusting for traditional risk factors (OR 1.353, p=0.031).

## Conclusions

Increased EAT thickness assessed by CMR is an independent risk factor for significant coronary artery stenosis in asymptomatic type 2 diabetes; however, the thickness was not associated with silent myocardial ischemia.

## Funding

None.

